# Pharmacokinetic study of tianeptine and its active metabolite MC5 in rats following different routes of administration using a novel liquid chromatography tandem mass spectrometry analytical method

**DOI:** 10.1007/s00210-017-1448-2

**Published:** 2017-12-12

**Authors:** Małgorzata Szafarz, Agnieszka Wencel, Krzysztof Pociecha, Filip A. Fedak, Piotr Wlaź, Elżbieta Wyska

**Affiliations:** 1 Department of Pharmacokinetics and Physical Pharmacy, Faculty of Pharmacy, Jagiellonian University Medical College, Krakow, Poland; 2 Department of Animal Physiology, Institute of Biology and Biochemistry, Faculty of Biology and Biotechnology, Maria Curie-Sklodowska University, Lublin, Poland

**Keywords:** Tianeptine, MC5 metabolite, Pharmacokinetics, Rats, Mass spectrometry

## Abstract

Tianeptine is an atypical antidepressant with a unique mechanism of action and recently it has been also reported that its major metabolite, compound MC5, possesses pharmacological activity similar to that of the parent drug. The current study aims to investigate the pharmacokinetics (PK) of both tianeptine and MC5 after intravenous or intraperitoneal administration of the parent drug as well as the metabolic ratio of MC5 in rats. To achieve these goals an LC-MS/MS method using the small sample volume for the quantitation of tianeptine and its active metabolite MC5 in rat plasma and liver perfusate has been developed and validated. Following an intravenous administration of tianeptine pharmacokinetic parameters were calculated by non-compartmental analysis. The average tianeptine volume of distribution at steady state was 2.03 L/kg and the systemic clearance equaled 1.84 L/h/kg. The mean elimination half-lives of tianeptine and MC5 metabolite were 1.16 and 7.53 h, respectively. The hepatic clearance of tianeptine determined in the isolated rat liver perfusion studies was similar to the perfusate flow rate despite the low metabolic ratio of MC5. Mass spectrometric analysis of rat bile indicated that tianeptine and MC5 metabolite are eliminated with bile as glucuronide and glutamine conjugates. Bioavailability of tianeptine after its intraperitoneal administration was 69%. The PK model with a metabolite compartment developed in this study for both tianeptine and MC5 metabolite after two routes of administration may facilitate tianeptine dosage selection for the prospective pharmacological experiments.

## Introduction

The challenge of treatment-resistant depression is one of the most significant in contemporary psychiatric therapy. It has been reported that only one-third of major depressive patients achieve remission after 3 months of monotherapy with antidepressants. The most common approaches for these patients include switching to another antidepressant of the same or different group or combining antidepressants with, e.g., atypical antipsychotics (Tobe and Rybakowski 2013). Tianeptine is an antidepressant in clinical use that has drawn much attention because of its unique mechanism of action. In contrast to tricyclic antidepressants (TCAs) and selective serotonin reuptake inhibitors (SSRI), tianeptine enhances the synaptic uptake of serotonin with minimal effects on that of norepinephrine and dopamine; nevertheless, its efficacy against depressive episodes is comparable with that of TCAs and fluoxetine (Uzbay 2008; Woo et al. 2013). It shows a faster onset of therapeutic effects against some depressive symptoms (cognitive and anxiety symptoms) as compared to other antidepressants and more importantly is effective in patients resistant to SSRI therapy (Woo et al. 2013). In addition, this drug improves learning and increases long-term memory in rodents, exerts a vigilance-enhancing effect in laboratory animals and improves cognitive functions in depressed patients (Klasik et al. 2011; Jaffard et al. 1991; Zoladz et al. 2008). Tianeptine promotes neuroplasticity by increasing the expression of genes of neuroplasticity factors (Reagan et al. 2007; Wlaź et al. 2011; Kuipers et al. 2013), reverses synaptic plasticity, dendritic atrophy, memory impairment, and neuronal loss under conditions of stress (Zhang et al. 2013). Moreover, it has also additional properties, including neuroprotection (Jantas et al. 2014), anticonvulsant activity (Ceyhan et al. 2005; Ceyhan et al. 2007), antinociceptive effect (Uzbay et al. 1999), inhibitory effects on ethanol withdrawal syndrome in rats (Uzbay et al. 2006), and anti-inflammatory effect in lipopolysaccharide-stimulated microglia (Slusarczyk et al. 2016). Recently, it has been revealed that tianeptine is a μ-opioid receptor (MOR) agonist and the hypothesis has been presented that precisely the MOR activation is the initial molecular event responsible for modulation of the glutamatergic system and for triggering many of known acute and chronic effects of tianeptine, including its antidepressant/anxiety actions (Gassaway et al. 2014).

Tianeptine is generally well tolerated by patients and causes less side effects compared with SSRI and TCAs (McEwen et al. 2010). In clinical trials, the most common adverse effects caused by this drug were gastrointestinal (nausea, constipation, abdominal pain) or CNS (headache, dizziness, change in dreaming) disturbances, which decreased in frequency with continued treatment. Tianeptine appears to have a lower propensity to cause sedative, anticholinergic, and cardiovascular effects than classical TCAs and it has only rarely been associated with hepatoxicity. The low incidences of anticholinergic effects, sedation, and cardiotoxicity make tianeptine particularly attractive for use in the elderly and in patients with previous alcoholism who are known to have increased sensitivity to the adverse effects of psychotropic drugs. Tianeptine has a wide therapeutic margin, and usually, overdosage has been associated with only minor transient adverse effects (Wilde and Benfield 1995; Wagstaff et al. 2001). However, a fatal case involving this drug has also been reported (Proenca et al. 2007).

**Fig. 1:**
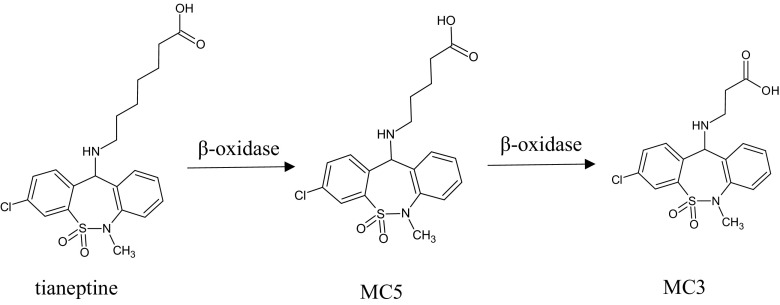
Metabolic pathways for tianeptine

Unlike other antidepressants, tianeptine is not primarily metabolized by the cytochrome P450 system. Instead, β-oxidation is the main metabolic pathway. Two major metabolites MC5 and MC3 are found in plasma and urine, respectively, and the behavioral effects of MC5 are comparable to those of tianeptine (Couet et al. 1990; Royer et al. 1988; Samuels et al. 2017) (Fig. 1).

In preclinical studies, tianeptine exerted beneficial effects in a number of behavioral models related to depression and anxiety in which rats and mice were used interchangeably. In pharmacological experiments in which rats were used as an animal model, this drug was administered either intravenously (i.v.) at the doses of 2.5, 5, 10, and 20 mg/kg (Bilge et al. 2012) or intraperitoneally (i.p.) at the doses of 2 and 10 mg/kg (Cavalla et al. 2015); 5, 10, and 15 mg/kg (Della et al. 2012); or at multiple (once a day for 21 days) doses of 10 mg/kg (Kuipers et al. 2013; Glombik et al. 2016). Identification of new molecular targets for tianeptine as well as the discovery that metabolite MC5 plays an active role in the behavioral effects of this drug provide a new perspective for interpreting tianeptine clinical and preclinical data. However, interpretation of pharmacological data might be difficult without the extensive knowledge about pharmacokinetic (PK) profiles of both tianeptine and MC5 following different routes of administration in laboratory animals commonly used in pharmacological experiments. Up to date, there is only a limited information on this subject in the available literature. Little is also known about the hepatic clearance and hepatic metabolic ratio of this drug in rodents. Tianeptine has been on the market for several decades and despite the fact that for most antidepressants therapeutic drug monitoring (TDM) has been recommended, there are no reports concerning the application of TDM for tianeptine in the clinic or the data dealing with tianeptine concentration-effect relationship in depressed patients. However, taking into account the recent reports regarding its new mechanisms of action and the contribution of metabolite MC5 to the observed pharmacological response after its administration, the availability of a fast and reliable analytical method for simultaneous determination of both compounds seems to be desirable not only from the therapeutic point of view but also due to tianeptine potential for the development of use disorder including abuse and addiction (Bence et al. 2016; Gupta et al. 2017).

Therefore, the purpose of this study was to determine PK of tianeptine and its active metabolite MC5 after two different routes of administration of the parent drug to rats, as well as its hepatic clearance, MC5 metabolic ratio, and bioavailability. To achieve these goals a simple, fast, and reliable analytical method for the simultaneous determination of tianeptine and MC5 metabolite in rats plasma and liver perfusate using small sample volumes was developed and validated.

## Experimental

### Chemicals and materials

Tianeptine sodium salt (purity ≥ 95%) was obtained from Cayman Chemical Company (USA), tianeptine metabolite MC5 sodium salt (purity ≥ 95%) was obtained from Toronto Research Chemicals (Canada). HPLC grade acetonitrile, formic acid, and ammonium formate were purchased from Merck (Germany) or Sigma Aldrich (USA). Pentoxifylline used as an internal standard was obtained from Sigma Aldrich (USA). Water was purified and deionized (conductivity below 0.1 μS) by the ultrapure water system HLP Smart 2000 (HydroLab, Poland). Chemicals used for the Krebs Hanseleit buffer preparation, as well as sodium taurocholate and glucose, were purchased from Sigma Aldrich (USA).

Control plasma, containing heparin as an anticoagulant, was obtained from adult male Wistar rats (Animal Facility of the Jagiellonian University Medical College, Krakow, Poland).

### Animals

Male Wistar rats, 13–15 weeks of age and weighing between 200 and 220 g, were used in all the experiments. They were kept under conditions of constant temperature (21–25 °C) and a relative humidity of approximately 40–65% with a standard light/dark cycle. Animals were housed in the stainless steel cages with suspended wire-mesh floors and had free access to water and the commercial rodent chow.

### Pharmacokinetic study in rats

Six rats were used in PK experiments, and 3 days prior to the experiment, rat jugular vein was cannulated allowing for the multiple blood sampling from a single animal. The experimental groups consisted of three rats each, and tianeptine dissolved in 0.9% sterile isotonic saline was administered i.v. at a dose of 1 mg/kg or i.p. at the dose of 10 mg/kg. Blood samples (approximately 300 μL) were collected to Eppendorf tubes containing heparin at 5, 15, 30, 60, 120, 240 and 480 min after dosing. Plasma was harvested by centrifuging at 3000 r.p.m. for 10 min and stored at − 30°C until bioanalysis. Plasma samples (100 μL) were further processed as described in “Sample preparation” section.

### Liver perfusion study

Rat livers were perfused in situ using PS-1 perfusion system from Harvard Apparatus-Hugo Sachs Electronics (HA-HSE). Rats were anesthetized by an intraperitoneal injection of ketamine/xylazine and after laparotomya ligature was placed around the vena cava inferior above a left renal vein. Heparin (ca. 500 units) was injected into the abdominal vena cava inferior and the portal vein was cannulated. Then, the abdominal vena cava inferior was transected and the perfusion buffer was pumped into the portal vein. In the next step, the chest was opened and a second cannulae was inserted through the right atrium into the thoracic vena cava inferior. Finally, the ligature around the abdominal vena cava inferior was tightened. The perfusions were conducted using freshly prepared Krebs Hanseleit buffer (118 mM NaCl, 4.7 mM KCl, 2.5 mM CaCl_2_, 1.19 mM KH_2_PO_4_, 1.18 MgSO_4_, and 25 mM NaHCO_3_, oxygenated with 95% O_2_/5% CO_2_, pH 7.4) with an addition of 1.2 g/L of glucose pumped through the liver at the constant flow rate equal 20–25 mL/min using a peristaltic pump. The temperature of the perfusion medium was thermostatically controlled at 37 °C. In each experiment, the common bile duct was cannulated with PE-10 tubing. After an initial stabilization period, within the first 15 min of perfusion, tianeptine was added (300 μg) into the buffer along with the 100 μL (20 mg/mL) of sodium taurocholate. Experiments were carried out in a recirculating manner with the total volume of perfusate equal 200 mL. The flow rate was sufficient to maintain the viability of the liver during the experiments, as determined by the macroscopic appearance, bile flow and in line measurement of oxygen consumption. Samples of bile were collected in pre-weighed Eppendorf vials and measured by gravimetric volume at the end of perfusion period, assuming a density of 1. Perfusate was collected from the reservoir at 5 min intervals up to 45 min. To test the stability of tianeptine under perfusion conditions, control experiments were performed with application of the same solution to the perfusion model without a liver resulting in no detectable degradation products of tianeptine.

### Preparation of stock solutions, calibration, and quality control samples

Two stock solutions of each tianeptine and MC5 metabolite were prepared separately by dissolving the reference compounds in methanol to give a final concentration of 1.0 mg/mL. They were further mixed and serially diluted with methanol in order to produce an appropriate calibration and quality control (QC) standards. The stock solution of pentoxifylline (internal standard, IS) was prepared at 1.0 mg/mL in the mixture of acetonitrile and water (50/50, *v*/*v*). Then IS working solution was prepared by diluting the stock solution with acetonitrile to give a concentration of 2.5 μg/mL. All stock and working solutions were stored at + 4 °C.

Calibration and QC samples were prepared by spiking 90 μL of pooled blank plasma or perfusate with a 10 μL of a working solution to produce the calibration curve points equivalent to 2000, 1000, 500, 250, 100, 50, 25, 10, and 1 ng/mL and QC standards at different concentrations along the calibration range (LLOQ at 1 ng/mL, low at 3 ng/mL, medium at 800 ng/mL, and high at 1800 ng/mL).

### Sample preparation

Samples were prepared using protein precipitation with acetonitrile. A portion (200 μL) of the working IS solution was spiked to 100 μL of plasma or perfusate sample and vortex-mixed for 90 s. Afterward, samples were centrifuged at 15000 r.p.m. for 5 min and the supernatant (100 μL) was transferred to the autosampler vials.

### LC-MS/MS quantification of tianeptine and its metabolite MC5 in biological samples

An Agilent 1100 system (Agilent Technologies, Germany) was used for solvent and sample delivery and chromatographic separation was achieved on an Aquasil C18 5 μm 3 × 100 mm analytical column (Thermo Scientific, USA). The mobile phase consisted of acetonitrile with an addition of 0.1% of formic acid (solvent A) and water with an addition of 4 mM of ammonium formate (solvent B). The mobile phase composition was 9:1 (A:B, *v*/*v*) and each LC-MS/MS run took 7 min. The flow rate was maintained at 400 μL/min and a sample volume of 5 μL was injected into the LC-MS/MS system.

Mass spectrometric detection was performed on an Applied Biosystems MDS Sciex (Concord, Canada) API 2000 triple quadrupole mass spectrometer with an electrospray ionization (ESI) in the positive ion mode. The tandem mass spectrometer was operated in the selected reaction monitoring (SRM) mode, and Q1 and Q3 quadrupoles were set at unit mass resolution. The ion source temperature was maintained at 450°C and the ionspray voltage was set at 5500 V. The mass transitions of the precursor/product ions were monitored at 437 → 292 *m*/*z* and 437 → 228 *m*/*z* for tianeptine, at 409 → 292 *m*/*z* and 409 → 228 *m*/*z* for metabolite MC5, and at 279 → 181 *m*/*z* for the IS. The Applied Biosystems Analyst version 1.4.2 software was used to control the LC-ESI/MS/MS system and to collect and treat the data.

### Method validation

Since perfusate is a simple matrix with strictly defined and fixed composition, the full method validation according to the EMA guidelines was performed only for the quantification of tianeptine and its MC5 metabolite in rat plasma (EMA 2012).

Precision and accuracy of the assay were assessed by performing replicate (*n* = 5) analyses of QC samples at different concentration levels on the same day (within-day) and on three different days (between-days). Accuracy (percent of recovery) was evaluated, using the following formula: [mean back calculated concentration/theoretical concentration] × 100. Precision was expressed as a percent relative standard deviation (RSD, CV%). The LLOQ based on QC samples was defined as the lowest analyte concentration that can be measured with the RSD ≤ 20% and the accuracy of 100 ± 20% on a day-to-day basis.

As the concentrations in selected samples were over 2000 ng/mL, the QC samples at fivefold of the highest level of calibration standard concentration were diluted by 10-fold and the dilution integrity was investigated by comparing the calculated concentration with nominal value.

An assessment of matrix effect (ME) was done by comparing the peak areas of analytes in extracted samples of blank plasma spiked with an analyzed compounds (post-extraction addition) with the corresponding peak areas obtained by injection of standard solutions at the appropriate concentration. For evaluation of the relative matrix effect, five different sources of rat plasma were used. Process efficiency (combined absolute matrix effect and recovery) was evaluated at three replicates of each QC samples. It was determined by comparing peak area obtained from extracted plasma samples with the standard solutions at the appropriate concentrations (Matuszewski et al. 2003).

The stability of analytes was determined in the battery of tests according to the appropriate guidelines. Namely, the bench top stability (for 2 h), freezer stability (at − 30°C for 90 days), freeze/thaw stability and post preparative autosampler stability (at 10°C up to 24 h). Samples were considered to be stable when 85–115% of the initial concentrations were found.

Additionally, the stability of standard working solutions was assessed after 90 days of storage at + 4°C.

### Pharmacokinetic analysis

Pharmacokinetic parameters were calculated by employing a non-compartmental approach, using Phoenix WinNonlin software v. 6.3 (Pharsight, USA). For i.v. administration, plasma concentration at time zero (*C*_0_) was back-extrapolated using log-linear regression, while the maximum concentration after i.p. administration (*C*_max_) and the time to *C*_max_ (*t*_max_) were obtained directly from the concentration-time data. The area under the mean plasma concentration versus time curve extrapolated to infinity (AUC_0-inf_) was estimated using the log/linear trapezoidal rule. AUMC_0-inf_ was estimated by calculation of the total area under the first-moment curve by combining the trapezoid calculation of AUMC_0-t_ and extrapolated area. The mean residence time (MRT) was calculated from AUMC_0-inf_/AUC_0-inf_. The terminal rate constant (*λ*_z_) was calculated by log-linear regression of the drug concentration data in the terminal phase and the terminal half-life (*t*_1/2_) was calculated as 0.693/*λ*_z_. The systemic clearance (CL) was estimated from the administered dose divided by AUC_0-inf_. The volume of distribution at steady state (*V*_ss_) was calculated from *D*_i.v._⋅AUMC/AUC^2^, where *D*_i.v_ is the dose administered i.v. in mg per kg of body weight. The concentration versus time profiles of the parent drug and metabolite following both routes of administration were also fitted simultaneously to the pharmacokinetic model with a metabolite compartment to obtain one set of parameters. During iteration process, the fraction of dose absorbed (*F*) was fixed to the value calculated using the following equation:

1 $$ F=\frac{AUC_{0-t(i.p.)}\bullet {D}_{i.v.}}{AUC_{0-t(i.v.)}\bullet {D}_{i.p.}} $$

where AUC_0-*t*_ is the area under the concentration versus time curve calculated to the last measured point and *D* is the dose administered.

Non-compartmental estimates of hepatic pharmacokinetic parameters were determined from the outflow concentration-time profiles. The hepatic extraction ratio (ER_H_) was calculated according to the following equation:

2 $$ {\mathrm{ER}}_{\mathrm{H}}=\frac{{\mathrm{CL}}_{\mathrm{H}}}{Q} $$

where CL_H_ is a hepatic clearance (per gram of liver) and *Q* is a perfusate flow (per gram of liver).

The apparent hepatobiliary clearance (CL_b,app_) was calculated as the cumulative amount of drug excreted in bile divided by the AUC_0-*t*_ of the drug in the perfusate. The metabolic ratio of MC5 metabolite was calculated by dividing the AUC_0-*t*_ of the metabolite by that of tianeptine.

### Statistical analysis

Statistical analysis was performed using Excel 2010 (Microsoft) and Statistica 10 (StatSoft, Tulsa, OK). Results were expressed as geometric means and 90% confidence intervals (CI) or arithmetic means ± SD.

## Results and discussion

### Analytical method and its validation

**Fig. 2:**
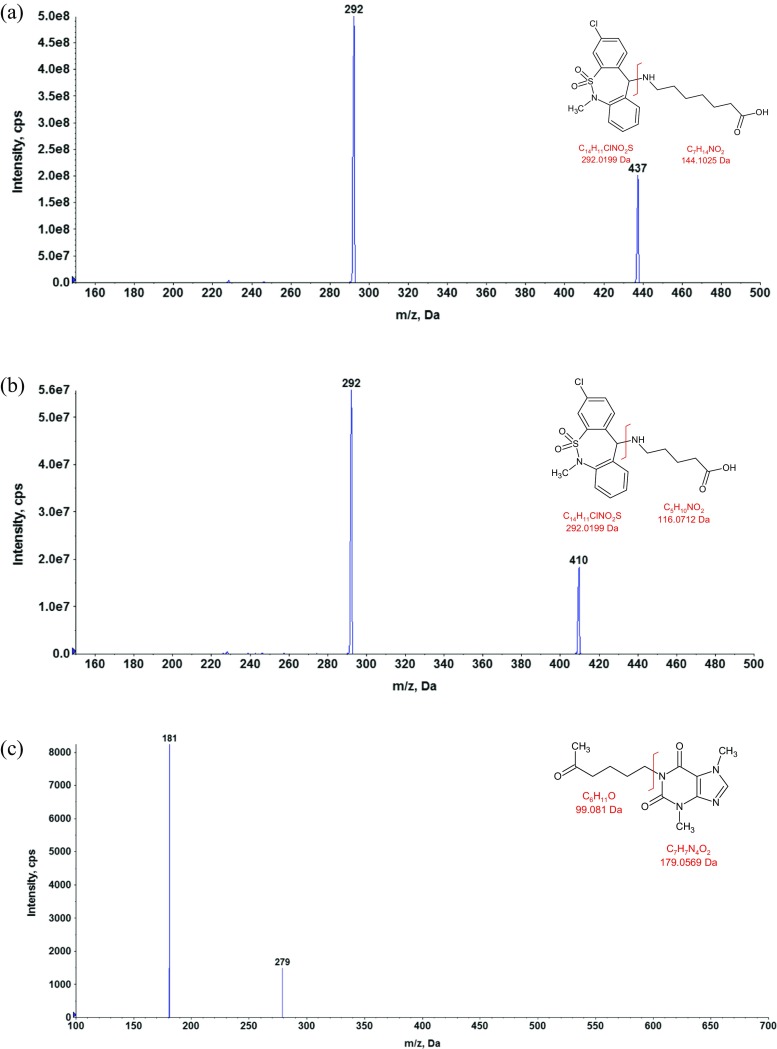
Product ion mass spectra of [M + H^+^]^+^ ions of tianeptine (**a**), metabolite MC5 (**b**), and IS (**c**) with the proposed fragmentation paths

**Fig. 3:**
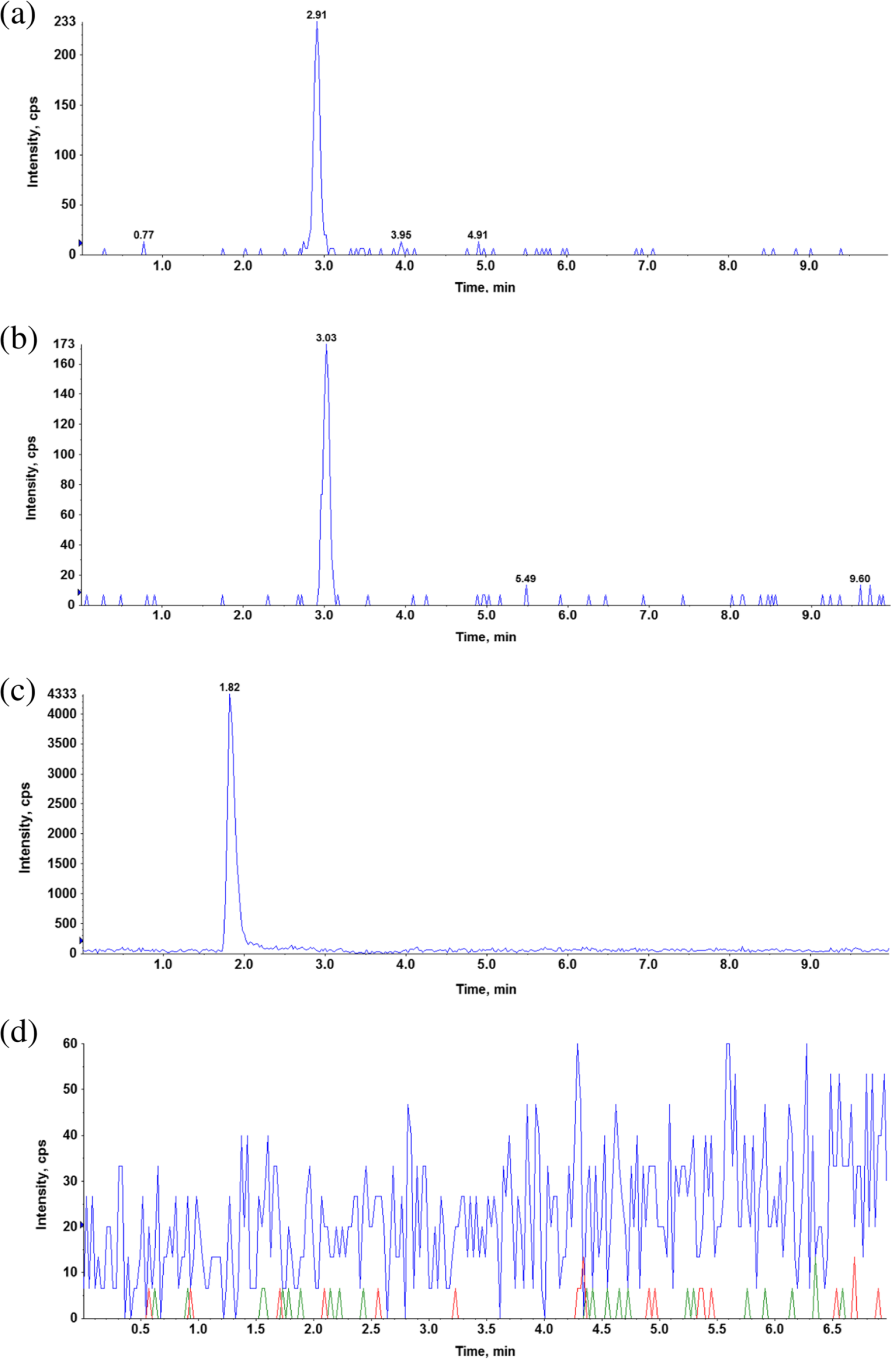
Representative chromatogram of QC sample at the LLOQ concentration (1 ng/mL). **a** Tianeptine. **b** Metabolite MC5. **c** Internal standard. **d** Blank sample

In order to avoid a time-consuming and tedious liquid–liquid extraction procedure, a simple and rapid one-step protein precipitation method was developed with acetonitrile selected as the optimal precipitation agent. Electrospray ionization operated in the positive ion mode was used for the LC-MS/MS analysis to provide the best sensitivity and selectivity. For the analyzed compounds the dominant ions in the Q1 spectra were used as the precursor ions to obtain Q3 product ion spectra. The resulting SRM transitions (precursor ion *m/z* → product ion *m/z*) for each analyte were as follows: 437 → 292 and 437 → 228 for tianeptine, 409 → 292 and 409 → 228 for MC5, and 279 → 181 for the IS. The first pair of ions was used as a quantifier and the second for the identification purpose (qualifier). Product ion mass spectra of [M + H]^+^ ions of tianeptine, metabolite MC5, and IS with the proposed fragmentation paths are presented in Fig. 2. An Aquasil C18 (3 × 100 mm, 5 μm) analytical column gave the best retention and baseline separation which in the case of tianeptine and MC5 metabolite was highly desirable to prevent the possibility of peak area contribution from one to the other since for the both analytes the most abundant product ion was at 292 *m*/*z*. A mobile phase consisting of acetonitrile with formic acid and water with ammonium formate was used at isocratic conditions. The representative extracted ion chromatograms (XIC) of a quality control sample at the concentration of 1 ng/mL (LLOQ) extracted from the supplemented plasma as well as a blank sample are shown in Fig. 3.

The specificity of the method was evaluated by analyzing blank plasma samples from six rats with no interfering peaks observed. The calibration curves were linear over the concentration range from 1 up to 2000 ng/mL of both analytes with correlation coefficients above 0.99 and consistent slope values when evaluated by weighted (1/*x* ^2^) least squares linear regression. The back calculated concentrations of calibrators are presented in Table 1. The lowest concentration on the calibration curve was chosen based on the expected concentrations in the biological matrices and the lower limit of quantification (LLOQ) was established at 1 ng/mL. LLOQ was accepted with a relative standard deviation of less than 20% and signal to noise ratio at least 20:1 (see Fig. 3). As seen in Table 2 the precision and accuracy values were within the acceptance criteria established by EMA for bioanalytical methods validation (EMA 2012).

**Table 1:** Back-calculated plasma concentrations of calibrators for tianeptine and metabolite MC5 (*n* = 5)

Nominal concentration (ng/mL)	Tianeptine			MC5		
	Back-calculated concentration (mean ± SD)	RSD (%)	Accuracy (%)	Back-calculated concentration (mean ± SD)	RSD (%)	Accuracy (%)
**1**	1.00 ± 0.002	0.22	99.83	1.00 ± 0.002	0.19	99.90
**10**	10.41 ± 1.00	9.7	104.14	10.34 ± 0.55	5.36	103.44
**25**	25.48 ± 2.05	8.03	101.92	24.93 ± 0.86	3.47	99.73
**50**	53.48 ± 2.57	4.80	106.96	53.68 ± 2.79	5.19	107.36
**100**	101.03 ± 8.94	8.85	101.03	99.30 ± 13.78	13.87	99.30
**250**	242.49 ± 12.44	5.13	96.99	238.45 ± 22.96	9.63	95.38
**500**	475.32 ± 31.17	6.56	95.06	473.80 ± 60.43	12.75	94.76
**1000**	1005.03 ± 15.62	1.55	100.50	1082.34 ± 51.35	4.74	108.23
**2000**	1967.83 ± 91.36	4.64	98.39	1912.68 ± 124.95	6.53	95.63

**Table 2:** Within-day and between-days accuracy (% of nominal concentration) and precision (%RSD) of tianeptine and MC5 in rat plasma (*n* = 5)

QC nominal concentration (ng/mL)	Tianeptine				MC5			
	Intra-day (%)		Inter-day (%)		Intra-day (%)		Inter-day (%)	
	Accuracy	Precision	Accuracy	Precision	Accuracy	Precision	Accuracy	Precision
1	99.78	10.8	100.19	8.03	89.67	0.23	100.96	11.49
3	107.29	3.34	105.48	5.12	91.97	2.84	98.11	8.15
800	100.25	9.54	96.63	8.96	90.48	3.69	93.10	7.59
1800	102.79	6.15	101.28	6.61	97.71	11.77	100.89	9.56

The relative ME was expressed as RSD (%) and the results showed a good repeatability from the samples coming from the pooled plasma as well as those coming from different animals and CV values were below 15%. The analysis of absolute ME did not show significant changes in ionization and the absolute recovery (process efficiency) was on average 60% for both analytes across the concentration range.

The stability of tianeptine and metabolite MC5 in rat plasma was investigated under a variety of storage and process conditions as described in “Method validation” section, and there were no stability related problems during the routine analysis of samples from pharmacokinetic studies.

### Pharmacokinetic study

Pharmacokinetic parameters, calculated based on the concentration-time profiles obtained after a single i.v. or i.p. administration of tianeptine, are presented in Table 3. Regardless of the route of administration, the elimination of MC5 metabolite was much slower than that of the parent drug with the mean terminal half-life for the MC5 metabolite equal 7.53 h as compared to 1.16 h for tianeptine. These results are in accordance with the outcomes of previously performed studies in rats and humans (Couet et al. 1990; Royer et al. 1988). After i.p. administration, tianeptine reached the peak concentration (*C*_max_) at the first sampling time (5 min), while metabolite MC5 attained *C*_max_ at 30 min after both routes of administration which is faster than the time reported in humans (2.96 ± 0.95 h) (Carlhant et al. 1990). Moreover, in contrast to the studies performed in humans, where the plasma metabolite concentrations were lower than those of the parent drug, in rats, the concentrations of MC5 after initial 30 min were similar or higher than those of tianeptine and an overall exposure to MC5 was 1.2 times higher as quantified by AUC than that of tianeptine regardless of the route of administration. In mice, which are the most commonly used animal model in behavioral studies, the conversion of tianeptine to MC5 metabolite seems to be even more efficient than in rats (Samuels et al. 2017; own unpublished data). Thus, in the light of recent reports regarding the pharmacological activity of metabolite MC5 being equal to that of the parent drug, the results of behavioral experiments performed on mice might be misleading (Samuels et al. 2017). Considering that the pharmacokinetic profile of tianeptine and its active metabolite in rats seems to be more in agreement with the results reported in humans as compared to the profile observed in mice it appears reasonable to argue that in this particular case rats could be better animal model than mice to be used in the further studies of tianeptine.

**Table 3:** Serum pharmacokinetic parameters calculated from concentration vs. time data (*n* = 3) of tianeptine and MC5 metabolite determined by non-compartmental analysis after a single intravenous or intraperitoneal administration of tianeptine to rats at a dose of 1 or 10 mg/kg, respectively. Data are presented as geometric mean (GM), 90% confidence interval (CI), and coefficient of variation (CV)

Parameter	Intravenous 1 mg/kg				Intraperitoneal 10 mg/kg			
	Tianeptine		MC5		Tianeptine		MC5	
	GM (90% CI)	CV%	GM (90% CI)	CV%	GM (90% CI)	CV%	GM (90% CI)	CV%
*C*_o_/*C*_max_ (mg/L)	1.27 (0.86–1.9)	23.64	0.117 (0.064–0.212)	22.16	6.65 (6.44–6.87)	1.3	2.02 (1.1–3.43)	22.35
*t*_max_ (h)	n/a		0.4 (0.054–2.89)	86.6	0.083	0	0.5	0
*λ*_z_ (h^−1^)	0.59 (0.25–1.42)	41.7	0.09 (0.085–0.099)	3.05	0.475 (0.332–0.68)	14.73	0.187 (0.07–0.498)	34.48
*t*_0.5λz_ (h)	1.16 (0.49–2.75)	64.34	7.53 (6.98–8.13)	3.05	1.46 (1.018–2.085)	14.03	3.69 (1.39–9.83)	42.29
*V*_z_(*V*_z_/*F*) (L/kg)	2.41 (0.91–6.4)	40.25	n/a	12.42	6.51 (4.03–10.53)	18.15	n/a	48.03
AUC_0-inf_ (mg∙h/L)	1954.02 (1411.02–2705.94)	19.44	2371.56 (1617.78–3476.46)	15.17	11,621.28 (9737.58–13,869.36)	7.05	14,415.96 (12,323.88–16,863.24)	6.35
CL/(CL/F) (L/h/kg)	1.84 (1.33–2.55)	21.39	n/a	15.52	3.1 (2.59–3.69)	7.17	n/a	5.98
*V*_ss_ (L/kg)	2.03 (0.89–4.62)	55.64	n/a		n/a		n/a	
MRT (h)	1.25 (0.66–2.36)	32.64	8.28 (6.53–10.5)	9.51	0.66 (0.51–0.87)	10.36	4.1 (1.83–9.24)	34.52

**Fig. 4:**
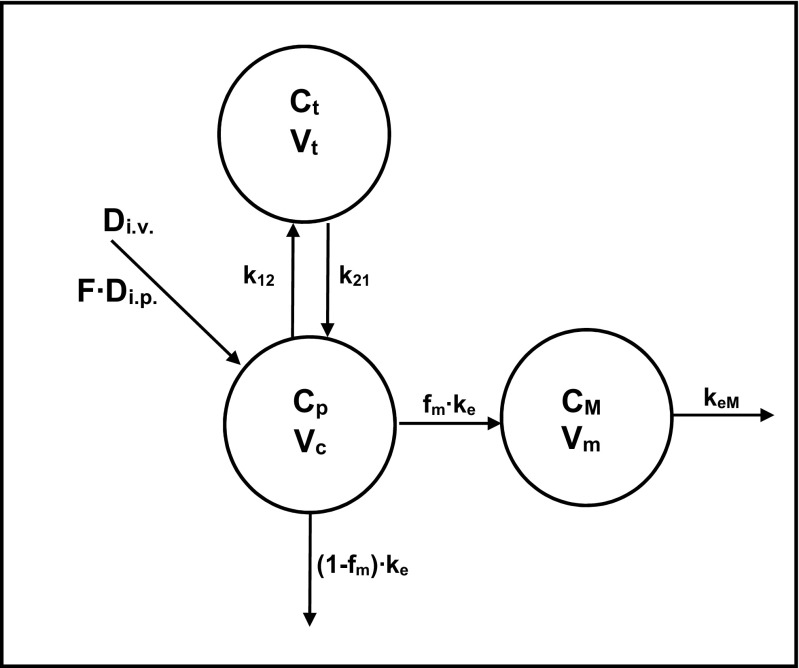
Pharmacokinetic model of tianeptine and its metabolite MC5 after two routes of administration of the parent drug. (*F* is fraction absorbed, *V*_c_, *V*_m_, and *V*_t_ are volumes of the central, metabolite, and tissue compartments, respectively, *k*_12_ and *k*_21_ are first order distribution and redistribution rate constants, *k*_e_ and *k*_eM_ are the first order rate constants for elimination of the parent drug and metabolite, respectively, and *f*_m_ is the fraction of the dose metabolized

**Fig. 5:**
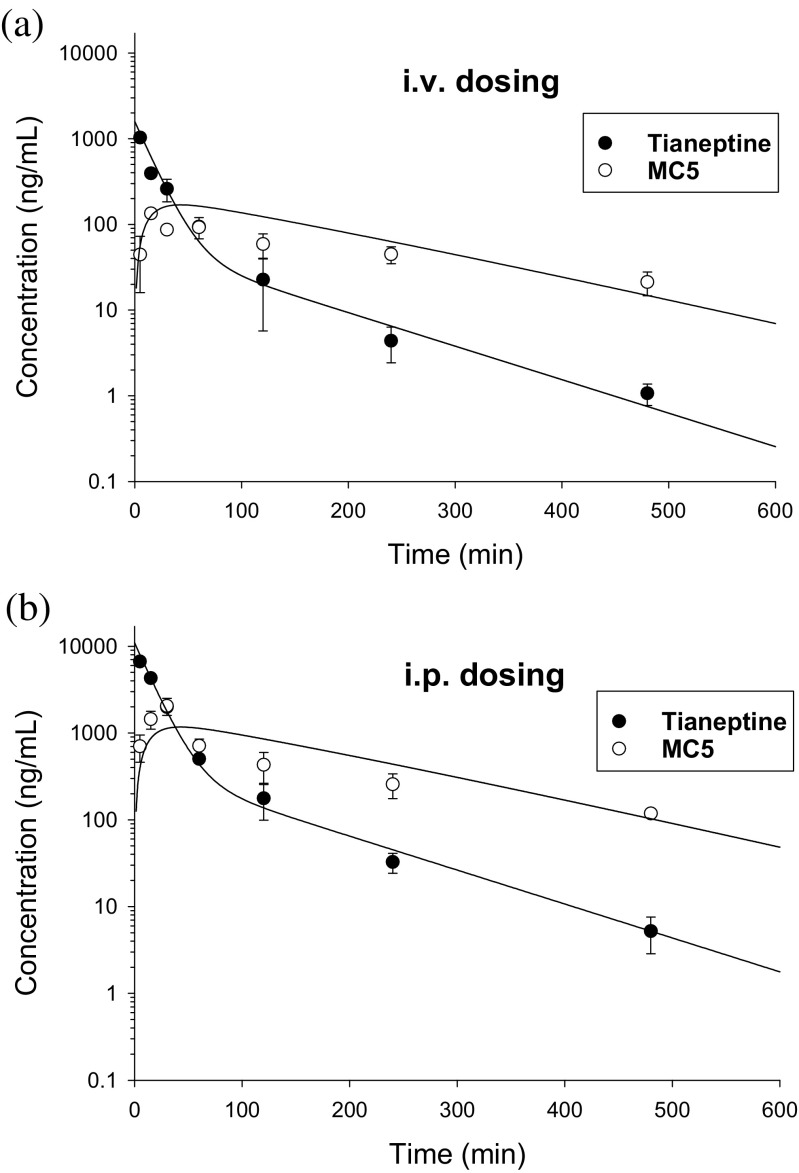
Measured (symbols) and pharmacokinetic model predicted (lines) concentration versus time profiles of tianeptine and its metabolite MC5 following intravenous (**a**) and intraperitoneal (**b**) administration of the parent drug to rats at the doses of 1 and 10 mg/kg, respectively

To facilitate the dosage selection for the prospective pharmacological experiments, the concentration versus time data of the parent drug and metabolite following both routes of administration were fitted simultaneously to the two-compartment pharmacokinetic model with an additional metabolite compartment (Fig. 4). The measured and pharmacokinetic model predicted concentration versus time profiles of tianeptine and its metabolite MC5 following i.v. or i.p. administration of the parent drug at the doses of 1 and 10 mg/kg, respectively, are presented in Fig. 5. It can be seen that the model very well captured the measured concentrations, what may be additionally confirmed by the low values of coefficients of variation (Table 4). Calculated based on the values of elimination rate constants, biological half-lives of tianeptine and MC5 metabolite were 0.25 and 1.66 h, respectively.

**Table 4:** Pharmacokinetic parameters of tianeptine and its metabolite MC5 following intravenous and intraperitoneal administration to rats estimated using the PK model with a metabolite compartment presented in Fig. 3

Parameter	Estimate	CV (%)
*V*_C_ (L/kg)	0.761	24.62
*V*_m_/*f*_m_ (L/kg)	2.971	15.6
*k*_e_ (h^−1^)	2.792	16.11
*k*_eM_ (h^−1^)	0.416	12.99
*k*_12_ (h^−1^)	0.504	39.92
*k*_21_ (h^−1^)	0.628	18.62
*F*	0.694 fixed	

The absolute bioavailability of tianeptine after intraperitoneal administration to rats calculated according to Eq. 1 equaled 69.4%. After i.p. administration, the first-pass effect was supposed to be less pronounced as compared to the oral route therefore these results once again confirm that in rats, metabolism of tianeptine is more efficient than in man where according to the available data, tianeptine is almost completely absorbed after oral administration (Royer et al. 1988; Wilde and Benfield 1995).

**Fig. 6:**
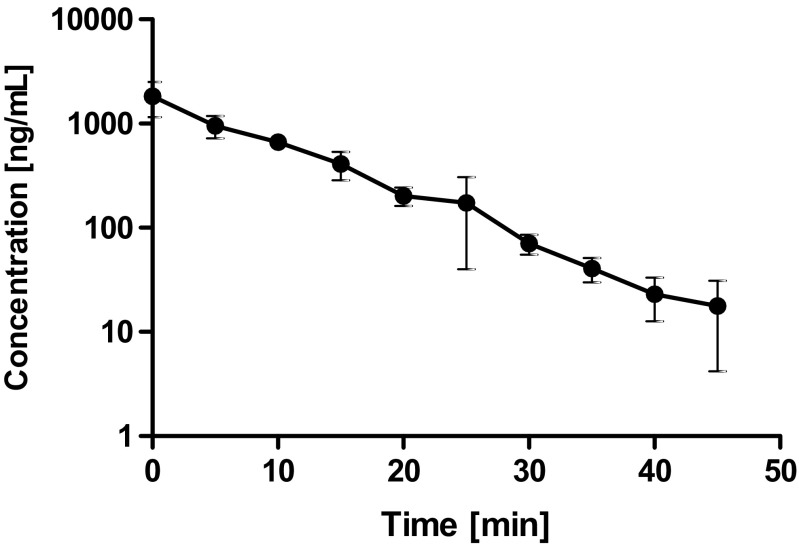
The perfusate concentration-time profile of tianeptine in the isolated perfused rat liver (mean ± SD; *n* = 3) after a single dose administration of the drug (300 μg)

**Fig. 7:**
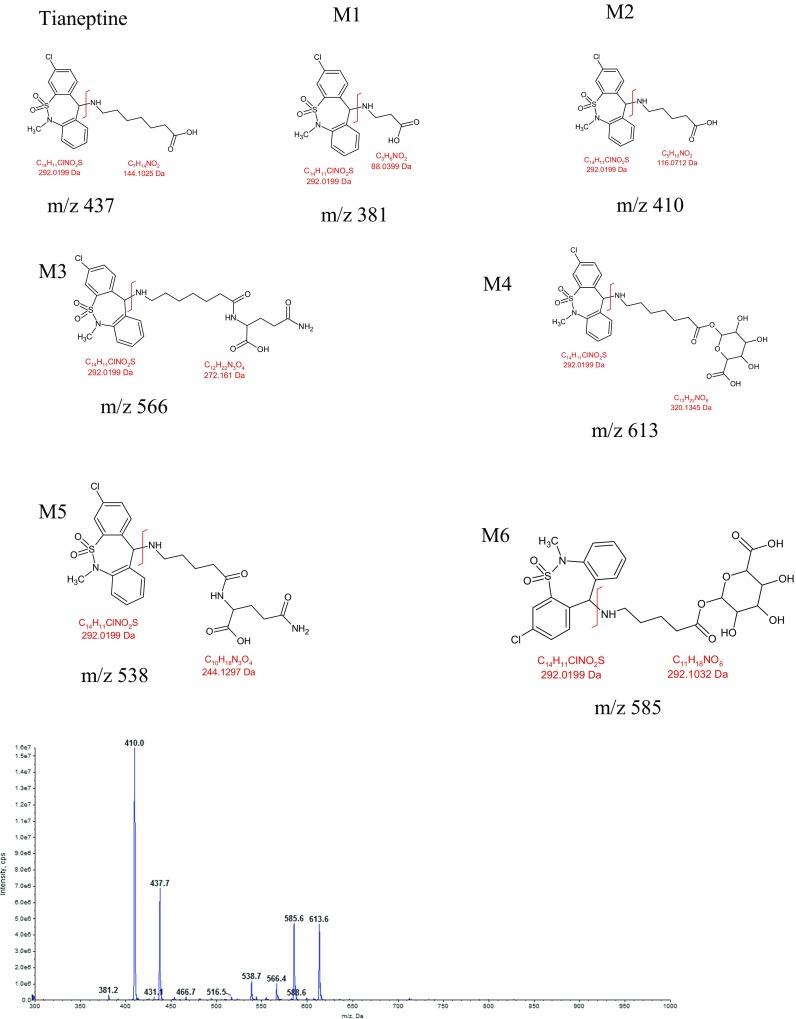
Mass spectra of rat bile sample scanned in the PI (precursor ion) 292 mode. *M*/*z* 437.7 — tianeptine; M1—metabolite MC3; M2—metabolite MC5; M3 and M4—conjugates of tianeptine with glutamine and glucuronic acid, respectively; M5 and M6—conjugates of metabolite MC5 with glutamine and glucuronic acid, respectively

Since liver is considered to be the main metabolic site for tianeptine, an additional experiments were performed using the system for isolated perfused organs of small animals in order to determine the hepatic clearance and hepatic metabolic ratio of this drug in rats. For the isolated rat liver perfusion (IPRL) studies, the total amount of drug added to the perfusion buffer equaled 300 μg, which considering the volume of the buffer used in each experiment (200 mL) gave the concentration of 1500 ng/mL. A similar *C*_0_ was reached after i.v. administration of tianeptine at the dose of 1 mg/kg (Table 3). The concentration of tianeptine in the perfusion buffer declined quite fast from the average value of 952 ng/mL at the first sampling time (5 min) to 17 ng/mL after 45 min (Fig. 6). The observed hepatic clearance was high and on average equal to the perfusion buffer flow rate. According to Eq. 2, it gave the high value of ER_H_ (104.55 ± 16.99%) further indicating that tianeptine in rats undergoes an extensive hepatic first-pass metabolism. In contrast to these findings, it has been reported that in humans, tianeptine is not subject to first-pass hepatic metabolism and its oral bioavailability equals to almost 100% (Wilde and Benfield 1995). The main tianeptine metabolites reported in the literature are MC5 (pentanoic acid derivative) and MC3 (propionic acid derivative) both being the products of β-oxidation. However unexpectedly, in the IPRL experiments, despite the high ER_H_, the metabolic ratio of MC5 was quite low (18.76 ± 8.11%) and the percent of MC5 eliminated into the bile up to 45 min of perfusion was only 3.07 ± 0.66% of the administered dose. At the same time the apparent hepatobiliary clearance was also low (0.2 ± 0.09 mL/min). Additionally, the percent of unchanged drug eliminated into the bile up to 45 min of perfusion was only 0.94 ± 0.33% of the administered dose. Since MC3 metabolite was undetected (based on the theoretical mass/charge ratio) in all analyzed samples, further analysis was performed in search of possible different routes of tianeptine metabolism in rats. Assuming that tianeptine, as well as its metabolites should give the same fragment ion *m*/*z* 292, the Q1 scans of rat bile were performed in the precursor ion mode. The major metabolites found in the analyzed samples are presented in Fig. 7. Beside previously identified MC3 and MC5 metabolites, different conjugates, mainly with glutamine and glucuronic acid, were also present what may explain the high ER_H_ value and the low metabolic ratio of MC5 observed in rats in the liver perfusion experiments.

## Conclusions

In this manuscript, pharmacokinetics of tianeptine and its active metabolite MC5 in rats following two routes of administration was studied. In order to achieve study goals, a sensitive and selective high performance liquid chromatography-tandem mass spectrometry method for the simultaneous determination of both compounds was developed. The validation of the method showed that the method is accurate and precise with the linear response of mass spectrometer in the plasma concentration range from 1 to 2000 ng/mL. This method may also be used in human studies as the transfer of an analytical method to a similar matrix, e.g., from rat to human plasma will require only partial validation. Pharmacokinetic analysis indicated that in rats, elimination of MC5 metabolite was much slower than that of the parent drug. In contrast to the results of the studies performed in humans, in the elimination phase, concentrations of MC5 were higher than those of tianeptine, although these differences are less pronounced when comparing MC5 concentrations in mice and humans. The results of ex vivo study indicated that in rats both MC5 metabolite and the parent drug are eliminated as glutamine and glucuronide conjugates. The proposed in this study pharmacokinetic model describing the concentration-time profiles of tianeptine and MC5 metabolite after two routes of administration may facilitate the dosage selection for the prospective pharmacological experiments.

In view of recent reports demonstrating the activity of MC5 metabolite being equal to that of tianeptine itself, the differences in the pharmacokinetics of these two compounds observed especially in the metabolism process between laboratory animals and man seem particularly important for the accurate interpretation of the results of pharmacological experiments.
